# Role of the MPTP in conditioning the heart – translatability and mechanism

**DOI:** 10.1111/bph.13013

**Published:** 2015-01-08

**Authors:** S‐B Ong, R K Dongworth, H A Cabrera‐Fuentes, D J Hausenloy

**Affiliations:** ^1^ The Hatter Cardiovascular InstituteUniversity College LondonLondonUK; ^2^ Institute of BiochemistryMedical SchoolJustus‐Liebig UniversityGiessenGermany; ^3^ Department of MicrobiologyKazan Federal UniversityKazanRussian Federation

## Abstract

Mitochondria have long been known to be the gatekeepers of cell fate. This is particularly so in the response to acute ischaemia‐reperfusion injury (IRI). Following an acute episode of sustained myocardial ischaemia, the opening of the mitochondrial permeability transition pore (MPTP) in the first few minutes of reperfusion, mediates cell death. Preventing MPTP opening at the onset of reperfusion using either pharmacological inhibitors [such as cyclosporin A (CsA) ] or genetic ablation has been reported to reduce myocardial infarct (MI) size in animal models of acute IRI. Interestingly, the endogenous cardioprotective intervention of ischaemic conditioning, in which the heart is protected against MI by applying cycles of brief ischaemia and reperfusion to either the heart itself or a remote organ or tissue, appears to be mediated through the inhibition of MPTP opening at reperfusion. Small proof‐of‐concept clinical studies have demonstrated the translatability of this therapeutic approach to target MPTP opening using CsA in clinical settings of acute myocardial IRI. However, given that CsA is a not a specific MPTP inhibitor, more novel and specific inhibitors of the MPTP need to be discovered – the molecular identification of the MPTP should facilitate this. In this paper, we review the role of the MPTP as a target for cardioprotection, the potential mechanisms underlying MPTP inhibition in the setting of ischaemic conditioning, and the translatability of MPTP inhibition as a therapeutic approach in the clinical setting.

**Linked Articles:**

This article is part of a themed section on Conditioning the Heart – Pathways to Translation. To view the other articles in this section visit http://dx.doi.org/10.1111/bph.2015.172.issue‐8

**Abbreviations**
ANT: adenine nucleotide translocase
CABG: coronary artery bypass graft
CsA: cyclosporin A
CypD: cyclophilin D
Drp1: dynamin‐related protein 1
GSK: glycogen synthase kinase
IPC: ischaemic preconditioning
IPost: ischaemic postconditioning
IRI: ischaemia‐reperfusion injury
LV: left ventricular
MI: myocardial infarct
MitoK_ATP_: mitochondrial ATP‐sensitive potassium channel
MPTP: mitochondrial permeability transition pore
OMM: outer mitochondrial membrane
OPA1: optic atrophy 1
PMI: perioperative myocardial injury
PPCI: primary percutaneous coronary intervention
RIC: remote ischaemic conditioning
RISK: reperfusion injury salvage kinase
ROS: reactive oxygen species
SAFE: survivor activating factor enhancement
STEMI: ST segment elevation myocardial infarction
VDAC: voltage‐dependent anion channel

Tables of Links

**Table:** 

TARGETS	
**Enzymes** *^a^*	**Ion channels** *^b^*
Akt (PKB)	MitoKATP, mitochondrial KATP channel, Kir6.2
ALDH2, aldehyde dehydrogenase 2	VDAC, voltage‐dependent anion channel
ERK1/2	**Transporters** *^c^*
F1F0 ATP synthase, c‐subunit	ANT, adenine nucleotide translocase
GSK, glycogen synthase kinase‐3β	Mitochondrial phosphate carrier, SLC25A3
HKII, hexokinase II	Sodium –hydrogen ion exchanger, SLC9
PKA	
PKC‐ε	
PKG

**Table:** 

LIGANDS
CsA, cyclosporin A
Diazoxide
Hydrogen peroxide
TNF‐α

These Tables list key protein targets and ligands in this article which are hyperlinked to corresponding entries in http://www.guidetopharmacology.org, the common portal for data from the IUPHAR/BPS Guide to PHARMACOLOGY (Pawson *et al*., 2014) and are permanently archived in the Concise Guide to PHARMACOLOGY 2013/14 (*^a,b,c^*Alexander *et al*., 2013a, 2013b, 2013c).

## Introduction

The mitochondria are crucial determinants of cardiomyocyte fate following an episode of acute myocardial ischaemia‐reperfusion injury (IRI). In particular, reperfusing acutely ischaemic myocardium induces the opening of the mitochondrial permeability transition pore (MPTP), an event that mediates cardiomyocyte death by uncoupling oxidative phosphorylation and causing mitochondrial swelling. Preclinical animal studies (Hausenloy *et al*., 2002; 2003) and initial proof‐of‐concept clinical trials (Piot *et al*., 2008; Chiari *et al*., 2014; Hausenloy *et al*., 2014) have demonstrated that the pharmacological inhibition of MPTP opening at the onset of reperfusion can attenuate cardiomyocyte death and reduce myocardial infarct (MI) size. The endogenous cardioprotective phenomenon of ischaemic conditioning, in which the heart is protected against MI by subjecting it to brief cycles of ischaemia and reperfusion, mediates its cardioprotective effect by recruiting many signalling pathways and preventing MPTP opening (Hausenloy *et al*., 2002; 2009a). In this paper, we review the role of the MPTP as a target for ischaemic conditioning, the potential mechanisms mediating MPTP inhibition in the setting of ischaemic conditioning and the translatability of MPTP inhibition as a therapeutic approach in the clinical setting.

## MPTP as a target for cardioprotection

The concept of a mitochondrial inner membrane pore opening to induce a transition in mitochondrial permeability was first proposed and characterized in seminal studies by Haworth and Hunter in the late 1970s (Haworth and Hunter, 1979; Hunter and Haworth, 1979a, 1979b). Despite intensive investigation, the molecular identity of the MPTP is still undefined, with transgenic animal studies excluding several putative components including the adenine nucleotide translocase (ANT) (Kokoszka *et al*., 2004), the voltage‐dependent anion channel (VDAC) (Baines *et al*., 2007), and more recently the mitochondrial phosphate carrier (SLC25A3; Kwong *et al*., 2014a, 2014b). Conversely, mitochondrial cyclophilin D (CypD) has been implicated as an important regulatory component of the MPTP (Baines *et al*., 2005; Basso *et al*., 2005), and most recently, the *c*‐subunit of the ATP synthase has been proposed to form the pore component of the MPTP in the inner mitochondrial membrane (Bonora *et al*., 2013; Giorgio *et al*., 2013; Alavian *et al*., 2014).

Pioneering studies by Crompton's group in the late 1980s were the first to implicate the MPTP as a critical determinant of cardiomyocyte death in the setting of acute IRI showing that ATP depletion, mitochondrial calcium overload, oxidative stress and phosphate levels were key inducers of MPTP opening, which could be inhibited by cyclosporin A (CsA; Crompton *et al*., 1987; 1988). Griffiths and Halestrap (1995) then made the crucial discovery that the MPTP remained closed during the index ischaemic period, and only opened in the first 2–3 min of reperfusion. We later confirmed that MPTP opening occurred primarily at the onset of reperfusion by showing that administering CsA solely at the onset of myocardial reperfusion limited MI size (Hausenloy *et al*., 2002). Crucially, we found that the cardioprotective effects of MPTP inhibition were completely lost if the MPTP inhibitor was administered after the first 15 min of myocardial reperfusion had elapsed (Hausenloy *et al*., 2003), emphasizing the importance of intervening in the first few minutes to prevent MPTP opening. This has defined a critical time window for employing MPTP inhibition and targeting myocardial reperfusion injury as a cardioprotective strategy, such that a therapeutic strategy designed to target reperfusion injury has to be administered prior to or at the immediate onset of reperfusion – this critical timing of the therapeutic intervention has had important implications in the translation of cardioprotection into the clinical setting (Hausenloy *et al*., 2011; 2013; Ovize *et al*., 2010).

## MPTP as target for ischaemic conditioning

Inhibition of MPTP opening at the onset of reperfusion has been shown to underlie the cardioprotection elicited by the endogenous phenomenon of ‘ischaemic conditioning’, a term encompassing the various approaches to myocardial ‘conditioning’ where the heart is rendered resistant to acute IRI by subjecting it to one or more cycles of brief ischaemia and reperfusion (Murry *et al*., 1986; Hausenloy, 2013). Experimental studies have demonstrated that MPTP opening is inhibited at the onset of reperfusion in hearts subjected to either ischaemic preconditioning (IPC, in which the heart is subjected to one of more brief cycles of ischaemia and reperfusion prior to the index ischaemia) (Murry *et al*., 1986; Hausenloy *et al*., 2002; 2004; Javadov *et al*., 2003) or ischaemic postconditioning (IPost, in which myocardial reperfusion following the index ischaemic event is interrupted by several short‐lived episodes of myocardial ischaemia) (Zhao *et al*., 2003; Argaud *et al*., 2005). Whether MPTP inhibition underlies the cardioprotection elicited by remote ischaemic conditioning (RIC) (Przyklenk *et al*., 1993; Hausenloy and Yellon, 2008), whereby the cardioprotective stimulus is applied to an organ or tissue away from the heart is not known.

### MPTP as a target for IPC

In 2001, Xu *et al*. (2001) first proposed that the MPTP may be a potential target of calcium‐induced preconditioning in isolated cardiomyocytes, although no direct experimental evidence of MPTP inhibition was provided. We then demonstrated that the preconditioning mimetic, diazoxide, a purported opener of the mitochondrial ATP‐sensitive potassium channel (MitoK_ATP_) channel, inhibits calcium‐induced MPTP opening in adult rat cardiac mitochondria (Hausenloy *et al*., 2002). Subsequent studies have confirmed MPTP inhibition at the time of reperfusion in several different settings of preconditioning (Javadov *et al*., 2003; Argaud *et al*., 2004; Hausenloy *et al*., 2004; Juhaszova *et al*., 2004).

### MPTP as a target for IPost

Experimental studies have also linked IPost cardioprotection to the inhibition of MPTP opening at the onset of reperfusion. In 2005, Ovize's research group (Argaud *et al*., 2005) were the first to demonstrate calcium‐induced MPTP inhibition in mitochondria isolated from IPost‐treated rabbit hearts (Bopassa *et al*., 2006; Fang *et al*., 2008). Importantly, our research group (Lim *et al*., 2007) have found that mice deficient in mitochondrial CypD were resistant to the infarct‐limiting effects of IPost confirming the importance of CypD in MPTP.

### MPTP as a target for RIC

The mechanism through which the cardioprotective signal applied to an organ or tissue remote from the heart activates pro‐survival signalling pathways in the heart is not clear. Wang *et al*. (2008) have shown that RIC *in vivo* using limb preconditioning generated a dialysate, which protected naïve perfused rabbit hearts against the myocardial IRI in terms of preserved outer mitochondrial membrane (OMM) integrity and maintained mitochondrial function. However, no studies have investigated directly whether the MPTP is a target for cardioprotection in the setting of RIC.

## How does ischaemic conditioning inhibit MPTP opening

**Figure 1:**
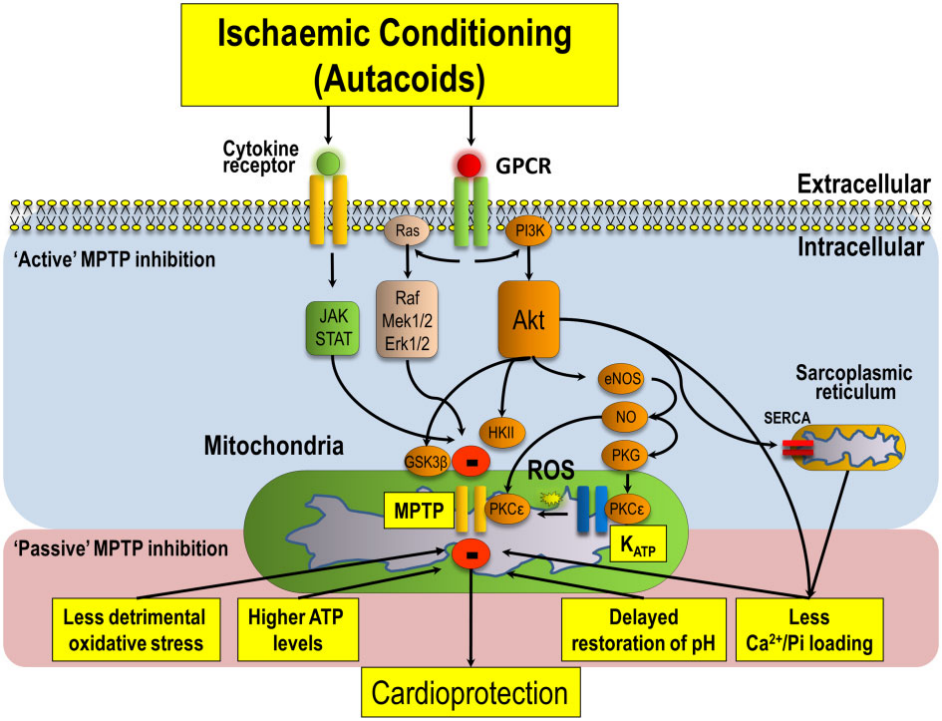
Reperfusion signalling pathways underlying ischaemic conditioning. The diagram provides a simplified scheme of some of the potential reperfusion signalling pathways linking ischaemic conditioning to the MPTP. These can be categorized into: (i) ‘Active MPTP inhibition’ (light blue shaded box): this includes those mechanistic pathways in which a signal transduction pathway has been implicated. This begins at the cardiomyocyte plasma membrane with the activation of the G‐protein coupled or cytokine receptor by autocoids such as adenosine, bradykinin or opioids, which result in the recruitment of complex signal transduction pathways many of which terminate on the mitochondria, and involve in some cases the translocation of protein kinases to the mitochondria. For the sake of clarity only the components of the RISK (PI3K‐Akt and MEK1/2‐Erk1/2), SAFE (JAK‐STAT) and the PKG pathways are shown on this diagram. These reperfusion salvage pathways have been shown to activate downstream mediators such as eNOS, GSK‐3β, HKII, PKC‐ε, the mitochondrial ATP‐dependent potassium channel (K_ATP_), which then mediate the inhibitory effect on MPTP opening. (ii) ‘Passive MPTP inhibition’ (purple shaded box): this includes mechanisms that result in MPTP inhibition as an indirect effect of ischaemic conditioning on factors that are known to induce MPTP opening at the time of myocardial reperfusion such as attenuating detrimental ROS production, preserving ATP levels, delaying pH correction at reperfusion, and reducing mitochondrial calcium and phosphate overload. Clearly, these two categories are not mutually exclusive and there may overlap, for example, both PKG and PI3K‐Akt have been reported to influence intracellular calcium regulation by promoting the uptake of calcium via SERCA into the sarcoplasmic reticulum [modified from Hausenloy *et al*., 2009b).

The actual mechanism through which the cardioprotective signal elicited by ischaemic conditioning mediates its inhibitory effect on MPTP opening at the time of myocardial reperfusion is not clear. A number of potential mechanisms have been proposed, and these can be broadly divided into two different categories (which may not be mutually exclusive) (as summarised in Figure 1):

(1)  Passive pathway – ischaemic conditioning modulates factors such as cellular energy status, mitochondrial calcium and phosphate accumulation, oxidative stress, and intracellular pH changes, which are known to affect MPTP opening susceptibility (Griffiths and Halestrap, 1995; Hausenloy and Yellon, 2003; Halestrap and Richardson, 2014);
(2)  Active pathway – ischaemic conditioning activates a signalling pathway, which then modifies MPTP opening susceptibility by either interacting with putative components of the MPTP, or by modulating the same factors alluded to in the ‘passive pathway’.

### Passive pathway of MPTP inhibition in ischaemic conditioning

#### Preserving mitochondrial energy during acute IRI

The cellular de‐energization and depletion of ATP induced by acute prolonged myocardial ischaemia contributes to the opening of the MPTP at the time of reperfusion. Murry *et al*. (1986) first proposed in their seminal IPC study that the preservation of myocardial ATP levels during ischaemia was critical to IPC cardioprotection. This mechanism was confirmed in several experimental studies, although linking this effect to MPTP inhibition has not been shown (Murry *et al*., 1990; Kobara *et al*., 1996; Fryer *et al*., 2000; Laclau *et al*., 2001).

#### Attenuating mitochondrial calcium overload during acute IRI

Intracellular acidosis during acute myocardial ischaemia results in intracellular calcium overload and subsequent mitochondrial calcium accumulation, which is exacerbated at the time of reperfusion and results in MPTP opening (Ruiz‐Meana *et al*., 2006). It has been suggested that by opening the ATP‐dependent mitochondrial potassium channel (MitoK_ATP_), IPC may reduce mitochondrial calcium overload during ischaemia and inhibit MPTP opening at reperfusion (Murata *et al*., 2001), although this mechanism has been questioned (Ardehali and O'Rourke, 2005; Hanley and Daut, 2005). Recent experimental studies have suggested that oxidative stress and the restoration of physiological pH may be more important inducers of MPTP opening at the onset of reperfusion than mitochondrial calcium overload (Kim *et al*., 2006; Ruiz‐Meana *et al*., 2006). Furthermore, genetic ablation of the recently discovered mitochondrial calcium uniporter was found to reduce the sensitivity to calcium‐induced MPTP opening, but did not have any effect on the susceptibility of the heart to acute IRI, again questioning the role of mitochondrial calcium overload as a trigger of MPTP opening at the time of reperfusion (Pan *et al*., 2013).

#### Attenuating oxidative stress production during acute IRI

Oxidative stress generated during acute myocardial ischaemia may prime the MPTP to open at the time of reperfusion (Robin *et al*., 2007), and the production of oxidative stress from the re‐energized electron transport chain in the first few minutes of reperfusion is a major factor for inducing MPTP opening. Experimental studies have shown that both IPC and IPost attenuate the production of oxidative stress at the time of reperfusion (Crestanello *et al*., 1996; Zhao *et al*., 2003; Sun *et al*., 2005; Clarke *et al*., 2008), although this effect has not yet been linked to MPTP inhibition. The actual mechanism through which IPC and IPost actually attenuate oxidative stress at the time of reperfusion is not clear. Halestrap's group (Pasdois *et al*., 2011) has postulated that IPC preserves the integrity of the OMM, thereby preventing loss of cytochrome *c* into the cytosol, thereby attenuating the production of oxidative stress and MPTP opening at reperfusion (Pasdois *et al*., 2011). More recently, Murphy's group (Chouchani *et al*., 2014) has shown that ischaemia results in the myocardial accumulation of the mitochondrial complex II substrate, succinate, which at the onset of reperfusion then provides a substrate load to complex II, which via reverse electron transport feeding to complex I, generates the oxidative stress observed in the first few minutes of reperfusion. Whether IPC confers its cardioprotective effect by attenuating the ischaemia‐induced accumulation of succinate, and IPost does so by antagonizing the production of oxidative stress from complex I at the onset of reperfusion, remains to be investigated.

#### Delaying intracellular pH correction at reperfusion

During the index ischaemia, the intracellular acidosis keeps the MPTP closed, and it is only in the first few minutes of reperfusion with the rapid restoration of intracellular pH that permits the MPTP to open at the onset of reperfusion (Yellon and Hausenloy, 2007). It has been proposed that both IPC and IPost may delay or modify the changes in intracellular pH at the onset of reperfusion such that there is a delay in the restoration of physiological pH thereby keeping the MPTP closed for the first few minutes of reperfusion (Cohen *et al*., 2007). Early studies had reported that IPC may attenuate the intracellular acidosis generated during ischaemia by either reducing myocardial glycogen depletion during index ischaemia (Asimakis *et al*., 1992; Wolfe *et al*., 1993) or by inhibiting the sodium ion–hydrogen ion exchanger at reperfusion (Xiao and Allen, 1999; 2000). Hori *et al*. (1991) first demonstrated, in 1991, that intermittent reperfusion induced transient acidosis and ameliorated myocardial stunning. Subsequent studies have confirmed that IPost delays the restoration of physiological pH at reperfusion, which prevents MPTP opening thereby allowing the activation of pro‐survival kinases such as Akt and ERK1/2 (the pH hypothesis) (Cohen *et al*., 2007; Fujita *et al*., 2007).

### Active pathways of MPTP inhibition in ischaemic conditioning

#### MitoK_ATP_ channel and the MPTP

MitoK_ATP_, a mediator of IPC, has been reported to exert cardioprotection by reducing cytosolic and mitochondrial calcium overload (Garlid *et al*., 1997; Liu *et al*., 1998; Garlid, 2000; Murata *et al*., 2001). More recently, Garlid has proposed a more direct mechanistic pathway to link MitoK_ATP_ channel opening with MPTP inhibition through PKG, mitochondrial PKC‐ε, the MitoK_ATP_ channel, matrix alkalinization, superoxide and hydrogen peroxide production from complex I (Costa *et al*., 2005; 2006; Jaburek *et al*., 2006; Costa and Garlid, 2008). Several issues remain unclear with this particular mechanistic pathway: whether mitochondria actually contain PKC‐ε and whether there are two pools of this protein are not clear; how cytosolic PKG is able to activate mitochondrial PKC‐ε is not known; how PKC‐ε inhibits MPTP opening is not clear; and finally whether this mechanistic pathway is actually operational in the first few minutes of reperfusion has not been demonstrated.

#### Reperfusion injury salvage kinase (RISK) and survival activation factor enhancement (SAFE) pathways and the MPTP

It is well‐established that the acute activation of cardioprotective kinase pathways (such as the RISK and SAFE pathways) at the onset of myocardial reperfusion, can limit MI size (Hausenloy and Yellon, 2004; 2007; Lecour, 2009; Hausenloy *et al*., 2011). Crucially, ischaemic conditioning has been reported to recruit these salvage kinase pathways at the onset of reperfusion and there is evidence linking these pathways to MPTP inhibition (Hausenloy *et al*., 2005; Breivik *et al*., 2011).

##### RISK pathway and the MPTP

All three forms of ischaemic conditioning (IPC, IPost and RIC) have been linked to the activation of the RISK pathway and in the cases of IPC and IPost the activation of this salvage kinase cascade has been shown to inhibit MPTP opening (Hausenloy *et al*., 2005; Bopassa *et al*., 2006; Davidson *et al*., 2006; Breivik *et al*., 2011). Experimental data have suggested that the salvage kinases of the RISK pathway such as Akt (Bijur and Jope, 2003), ERK1/2 (Baines *et al*., 2002) and PKG (Costa *et al*., 2005) may translocate to mitochondria, and in some cases, the mitochondrial translocation has been linked to MPTP inhibition, although the actual mechanisms are not known. The link between these cardioprotective kinases translocating to mitochondria and MPTP inhibition has been questioned, with the proposal that IPC inhibits MPTP opening by attenuating oxidative stress (Clarke *et al*., 2008). The mechanism through which RISK pathway mediates its inhibitory effect on MPTP opening is unclear although it may do so through the activation of downstream mediators such as PKG, glycogen synthase kinase (GSK)‐3β or hexokinase II (HKII).

#### PKG and the MPTP

The activation of the Akt component of the RISK pathway is known to recruit the eNOS‐NO‐sGC‐cGMP‐PKG pathway and through this cascade (Garlid *et al*., 2004; Garcia‐Dorado *et al*., 2009) the RISK pathway may inhibit MPTP opening. This pathway appears to be mediated through the translocation of PKG to the OMM where it inhibits MPTP opening (see MitoK_ATP_ channel and the MPTP).

#### GSK‐3β and the MPTP

An important downstream target of the Akt and Erk1/2 components of the RISK pathway is GSK‐3β, a protein kinase, which regulates a variety of cellular processes including apoptosis, growth and metabolism (Cohen and Frame, 2001). The phosphorylation and inactivation of GSK‐3β has been linked to cardioprotection by IPC and IPost (Tong *et al*., 2002; Gomez *et al*., 2008), although not all studies have been in agreement (Nishino *et al*., 2008). Sollott's group (Juhaszova *et al*., 2004) provided comprehensive *in vitro* evidence suggesting that the phosphorylation and inactivation of mitochondrial GSK‐3β with MPTP inhibition was the underlying mechanism for a diverse array of cardioprotective strategies. However, the mechanism through which mitochondrial GSK‐3β inhibition actually mediates MPTP inhibition is unclear. Nishihara *et al*. (2007) have reported that GSK‐3β associated with ANT in IPC‐treated hearts, but the ANT is no longer considered to be an essential component of the MPTP (Kokoszka *et al*., 2004). A more recent study has suggested that GSK‐3β inhibition, allows the dephosphorylation of the OMM protein, VDAC, which prevents the entry of adenine nucleotides into mitochondria, which would be expected to facilitate mitochondrial depolarization and reduce mitochondrial calcium accumulation and reactive oxygen species (ROS) production during myocardial ischaemia thereby preventing MPTP opening at the time of reperfusion (Das *et al*., 2008). Whether this mechanism actually operates in the setting of IPC and IPost remains to be investigated.

#### HKII and the MPTP

Another downstream mediator of the Akt component of the RISK pathway is the glycolytic enzyme HKII, the mitochondrial translocation of which has been implicated in IPC cardioprotection (Smeele *et al*., 2011), as reviewed in Zuurbier *et al*. (2009). A variety of different mechanisms have been proposed to explain the cardioprotective effect of mitochondrial HK II including the maintenance of OMM integrity during acute IRI (stabilizing the mitochondrial membrane potential and preventing the release of cytochrome *c*), attenuating ROS production, improving glucose‐induced insulin release, preventing acidosis through enhanced coupling of glycolysis and glucose oxidation, and inhibition of fatty acid oxidation (see Nederlof *et al*., 2014). Again the interplay between HK II and MPTP inhibition in the context of IPC or IPost remains unclear.

##### SAFE pathway and the MPTP

Recruitment of the SAFE pathway at the time of reperfusion, which includes TNF‐α and STAT‐3, has been associated with both IPC and IPost cardioprotection (Lacerda *et al*., 2009; Lecour, 2009). The activation of the SAFE pathway at the onset of reperfusion has also been linked to MPTP inhibition (Smith *et al*., 2006; Boengler *et al*., 2010), although the mechanism for this effect is not clear. Recent experimental data have suggested that STAT3 may actually reside in the mitochondria (Wegrzyn *et al*., 2009), but the mechanism through which it inhibits MPTP opening is not known. Again, whether this pathway operates at reperfusion in the setting of IPC or IPost has not been investigated.

#### Mitochondrial morphology and the MPTP

Mitochondria are dynamic structures capable of changing their morphology by undergoing either fusion to generate an elongated phenotype [regulated by the mitochondrial‐fusion proteins (mitofusins and optic atrophy 1, OPA1) ] or fission to form fragmented mitochondria [regulated by the mitochondrial‐fission proteins (dynamin‐related protein 1, Drp1; human fission protein 1) ] (Ong and Hausenloy, 2010; Hall and Hausenloy, 2012; Ong *et al*., 2013; Hall *et al*., 2014). Recent experimental data suggest that mitochondria undergo fission and MPTP opening in response to acute IRI, and genetic or pharmacological inhibition of mitochondrial fission inhibits MPTP opening and attenuate cell death (Ong *et al*., 2010). This initial data suggested a link between mitochondrial morphology and MPTP opening susceptibility, with inhibiting mitochondrial fission induced by acute IRI preventing MPTP opening at reperfusion.

Interestingly, some of the cardioprotective kinases, which have been linked to MPTP inhibition in the setting of ischaemic conditioning such as PKA (Sanada *et al*., 2004) and Akt, have been reported to modulate mitochondrial morphology through the phosphorylation of Drp1 (Chang and Blackstone, 2007; Cribbs and Strack, 2007) and OPA1 (Parra *et al*., 2014) respectively. A recent experimental study has shown that pharmacological preconditioning with nitrites inhibits mitochondrial fission through PKA‐induced phosphorylation and inhibition of Drp1 (Kamga *et al*., 2014). Furthermore, Akt has also been recently shown to modulate mitochondrial morphology, but whether it does so in the context of ischaemic conditioning is not known (Ong *et al*., 2014; Parra *et al*., 2014). Finally, whether ischaemic conditioning protects the heart against acute IRI by inhibiting mitochondrial fission and MPTP inhibition has not been investigated.

#### Aldehyde dehydrogenase‐2 and the MPTP

Mitochondrial aldehyde dehydrogenase 2 (ALDH2) has also been identified as a key enzyme involved in cytoprotection in the heart (Chen *et al*., 2008; Budas *et al*., 2009; 2010; Gong *et al*., 2013). ALDH2 is one of the 19 members of the ALDH gene family, which mediates the oxidation and detoxification of reactive aldehydes in different organs and cell types (Vasiliou and Nebert, 2005). ALDH2 is encoded in the nucleus and imported into the mitochondrial matrix, because of a 17‐amino acid N‐terminal mitochondrial localization sequence (Vasiliou and Nebert, 2005). PKC‐ε has been shown to phosphorylate ALDH2 *in vitro*, resulting in an increase in ALDH2 catalytic activity (Chen *et al*., 2008). At least two phosphorylation sites have been identified by MS, including Thr^185^ and Thr^412^ and possibly, Ser^279^ (Chen *et al*., 2008). Pretreatment with the ALDH2 agonist, N‐(1,3‐benzodioxol‐5‐ylmethyl)‐2,6‐dichlorobenzamide (Alda‐1) in an *in vivo* rat model of acute MI reduced cardiac damage (Chen *et al*., 2008). Ethanol preconditioning has also been demonstrated to promote activation and translocation of PKC‐ε into cardiac mitochondria where it interacts with ALDH2 (Budas *et al*., 2010). ALDH2 mediates detoxification of reactive aldehydes and bioactivation of nitroglycerin (see Budas *et al*., 2009). Upregulation of mitochondrial ALDH2 activity can also inhibit MPTP opening and thus induce cardioprotective outcomes (Li *et al*., 2010), although the exact mechanism remains unclear.

## Translatability of MPTP inhibition as a therapeutic approach

The majority of experimental studies have reported MPTP inhibition using CsA to be an effective cardioprotective strategy. However, there have been some neutral studies using *in vivo* MI models in rats (De *et al*., 2013) and pigs (Karlsson *et al*., 2010). The reason for these discrepant results is not clear (Hausenloy *et al*., 2012), although there are several potential factors including: (i) the presence of other types of cell death, such as apoptosis and programmed cell necrosis (or necroptosis) in the myocardium after acute IRI (Luedde *et al*., 2014) – in addition whether these other forms of cell death are attenuated by ischaemic conditioning needs to be determined; (ii) MPTP opening is not the only contributor to necrotic cell death following acute IRI (Garcia‐Dorado *et al*., 1997; Ruiz‐Meana *et al*., 2009); and (iii) MPTP opening may not be a target for cardioprotection following relatively short episodes of myocardial ischaemia (Ruiz‐Meana *et al*., 2011).

### MPTP as a therapeutic target in co‐morbid conditions

Given the potential confounding effects of factors such as age, diabetes, left ventricular (LV) hypertension, on cardioprotection (Ferdinandy *et al*., 2007; 2014), it is important to determine whether MPTP inhibition using CsA is effective in these settings. In this regard, Huhn *et al*. (2010) have reported that the pre‐diabetic Zucker obese rat was resistant to the MI‐limiting effects of CsA administered at the onset of reperfusion. In contrast, CsA‐cardioprotection was preserved in the aged murine heart subjected to acute IRI (Peart *et al*., 2014), suggesting that the modulation of CsA‐cardioprotection may depend on the particular confounding factor.

### MPTP inhibition as a therapeutic strategy in the clinical setting

#### Acute myocardial infarction

The first study to investigate the MPTP as a therapeutic target in the clinical setting of acute MI, was by Piot *et al*. (Piot *et al*., 2008), who randomized 58 patients presenting with an acute ST segment elevation myocardial infarction (STEMI) to receive either a single intravenous bolus of either CsA (2.5 mg/kg) or placebo 10 min prior to primary percutaneous coronary intervention (PPCI). In those patients who received CsA therapy, MI size was reduced by 40% when compared to placebo control. In a follow‐up study, it was demonstrated that MI size was reduced and there was less adverse LV remodelling on cardiac MRI scans performed at 5 days and 6 months (Mewton *et al*., 2010). However, in STEMI patients treated by thrombolytic therapy, CsA did not reduce MI size (Ghaffari *et al*., 2013). The reason for this discrepant finding is not clear, although thrombolytic therapy by inducing gradual reperfusion may reduce the magnitude of myocardial reperfusion injury.

The ongoing CYCLosporinE A in Reperfused Acute Myocardial Infarction (CYCLE) (http://www.ClinicalTrials.gov NCT01650662) and Cyclosporine and Prognosis in Acute Myocardial Infarction Patients (CIRCUS) (http://www.ClinicalTrials.gov NCT01502774) multi‐centre randomized clinical trials are currently investigating CsA therapy in PPCI‐treated STEMI patients.

#### Cardiac bypass surgery

Coronary revascularization by coronary artery bypass graft (CABG) surgery, subjects the heart to acute global IRI as the heart is put onto and taken off cardiopulmonary bypass. The extent of perioperative myocardial injury (PMI) can be quantified by measuring the release into blood of cardiac enzymes, such as creatine kinase and Troponin T or I, the release of which has been linked to worse clinical outcomes. We have recently found that administering a single dose of CsA prior to CABG surgery reduced the extent of PMI in CABG surgery patients (Hausenloy *et al*., 2014). Another study has shown beneficial effects of CsA in patients undergoing aortic valve surgery (Chiari *et al*., 2014).

## Need for more specific MPTP inhibitors

Although the discovery that MPTP opening could be inhibited by targeting CypD using CsA has allowed us to investigate the role of the MPTP as a mediator of acute IRI, CsA is not without its problems (see Hausenloy *et al*., 2012). The main concern is the specificity of CsA for the MPTP, given that it inhibits other cyclophilins and also suppresses activity of the phosphatase, calcineurin. In this regard, experimental animal studies have reported cardioprotective effects with CsA through mechanisms independent of MPTP inhibition and instead mediated by calcineurin inhibition (Weinbrenner *et al*., 1998; Cereghetti *et al*., 2008), cell volume regulation or cyclophilin A inhibition. As such, more novel and specific MPTP inhibitors need to be discovered if MPTP inhibition is going to be widely used as a therapeutic approach for cardioprotection. Knowing the identity, i.e., the components, of the MPTP should facilitate this process.

## Summary and conclusions

The opening of the MPTP at the onset of myocardial reperfusion is a critical determinant of myocardial reperfusion injury. Although the mechanism(s) underlying cardioprotection by ischaemic conditioning remains unclear, evidence suggests that it prevents MPTP opening at the reperfusion by modulating factors known to MPTP opening at this time, such as cellular energetic status, mitochondrial Ca^2+^ overloading, ROS production, and rapid pH correction either directly or indirectly via known cardioprotective signalling pathways. The identification of the molecular pore of the MPTP should provide further insight into unravelling the signalling pathway involved in inhibition of the MPTP and further enhance the translatability of MPTP inhibition to the clinical setting.

## Conflict of interest

None.
